# Notes and News

**Published:** 1889-03-16

**Authors:** 


### Notes and News.

Collected and Communicated.

*Tommy Traddles* (threateningly): "I'll tell my father on you."—*Willie Waffles:* "What do I care for your father? He can't hurt me."—*Tommy Traddles:* "He can't, can't he? My father is a doctor."

The annual meeting of the Royal Hospital for Women and Children was held last week, the Lord Mayor presiding. With an increased income last year, it extended its sphere of usefulness, and treated 450 in and 6,942 out-patients. To use its wards, however, to their fullest extent, an additional income of about £2,000 a year is still required.

We have received an excellent report of the Lockhart Hospital, Lanark. All details of expenditure are clearly given, so are details of the different cases; and the results bear strongly in favour of the skill and care of the doctors, and the matron-nurse, Miss McRae. The patients numbered 121 last year, and the expenditure was £319, leaving a balance in hand of £85. There is a dispensary connected with the hospital, which treated 560 patients last year.

The next meeting of The Hospitals Association will be held at the Middlesex Hospital, on Wednesday, 20th inst., when a paper on a deeply-interesting subject will be read by Mr. T. Ryan, secretary of St. Mary's Hospital, Paddington. The title of the paper is: "The Conveyance of Injured Persons to the Metropolitan Hospitals: Defects in the Existing System, and Proposals for their Removal." Sir John Heron Maxwell, Bart., will preside. An influential committee has been formed to carry the scheme into effect.

James Hayward, who was indicted by the Charity Organisation Society for obtaining money under false pretences, has been found not guilty. It seemed that some gentlemen were desirous of founding a hospital for persons in an advanced stage of consumption, and the defendant was employed to canvass for subscriptions. Subsequently the honorary secretary ceased to employ him, and advertised the fact in two of the daily newspapers. The case on the part of the prosecution was that, after he had ceased to be employed, the defendant collected some subscriptions, and that he had no authority to do so. The thanks of all are due to the Charity Organisation Society for having brought this matter before the public.

The secretary of the Royal Berkshire Hospital placed a good report before the annual meeting of the committee on Feb. 26th. The number of patients increases yearly, but unluckily the subscribers do not increase in proportion, and the hospital pleads a serious deficit This, however, is the jubilee year of the hospital, and no doubt the inhabitants of Reading will rise to the occasion, and make an effort to increase the support given to the hospital, and to wipe off the existing debt. Over 1,000 in-patients were treated last year, and over 1,500 out-patients.

At the late annual meeting of the Southport Infirmary, at which the Mayor presided, a deficiency of over £300 was acknowledged. This is very much to be regretted, as new buildings are badly needed. At present each nurse has to attend to 14 patients, though the average number of patients to a nurse in most hospitals is six; yet till further accommodation is provided for the nurses there is no room to increase their number. There ought to be more annual subscriptions forthcoming for the infirmary in a town like Southport. That all economy is practised at the infirmary is proved by the fact that each occupied bed costs but £43 9s. 10d. per annum.

There are about half-a-dozen too energetic people in the hospital world, who are always overdoing it with their reports and appeals. It would tire the patience of Job himself, if seated in the editorial chair, to receive every week some notice for insertion of an institution which is fairly well off, and ably supported by the dwellers in its neighbourhood. Because many of our London hospitals, and several of our country ones, are always in difficulties and troubled by debts, the smaller institutions think they ought also to be in the same condition. Cannot the secretaries see that it is just as commendable for a hospital to pay its way as for an individual to do so? When an institution has a balance to announce, it should announce it with pride, and not take pleasure in being a debtor and a beggar. The energetic secretary also overdoes it when he sends a printed form headed: "The following table will show at a glance the great progress made by the institution since my (!) appointment, twenty years ago."

The Annual Report of Leeds Infirmary, submitted to the Governors on March 4, tells a tale of increased usefulness, and of yet further necessity for growth. The total number of in-patients had reached 4,943. Of the beds occupied, the number was 285, as against 275 in 1887. The rate of mortality, excluding cases where patients had died within forty-eight hours after admission, had been 3·42 per cent. One great feature of the year had been the opening of the Ida Semi-Convalescent Hospital at Cookridge, provided by the munificence of Mr. John North. That institution contained 42 beds, which had been almost constantly occupied. The benefits conferred on the infirmary patients had quite equalled expectation, and had been of the greatest value. For the Ida Hospital the furnishing had cost £1,470, and the maintenance £948; and this had brought the expenditure of the infirmary for the year to £18,757, as against £16,513 in 1887. The average cost per patient per week at the infirmary had been 17s. 11¼d., or, inclusive of fittings, 19s. 6½d. At the Ida Hospital the cost of treatment, maintenance, and nursing had been 15s. 9d. per week; while the cost of each out-patient to the infirmary had been 1s. 3d. The total income had been £17,430, as against £17,339 in 1887, showing an almost stationary income. A note is appended to the conclusion of the report, pointing out why the cost per patient varies in different hospitals. Of course, till the managers of all hospitals agree to adopt a common basis upon which to calculate the cost of patients, this financial discrepancy will be a stumbling-block to many outsiders.

THE following vacancies are announced this week, the amount of salary in each case being given after the name of the institution :—

**Resident Medical Officers.**

Royal Victoria Hospital, Bournemouth. House Surgeon.—£100, board, etc.
West London Hospital, Hammersmith. House Surgeon.—Board, etc.
Hospital for Women, Soho Square. Two Clinical Assistants.

**Non-resident Medical Officers.**

Dental Hospital of London. Assistant Dental Surgeon.
Middlesex Hospital. Fourth Assistant Physician.
Woodhouse Grove School, Leeds. Medical Officer.
University of Glasgow. Four Examiners in Medicine.—£30.
King's College, London. Professor of Forensic Medicine.

---

WIRRAL HOMŒOPATHIC HOSPITAL governors look back on 1888 as a specially healthy and prosperous year. They gather from various statistics that the year just closed is the most healthy we have experienced for a long period, and in proof, their patients numbered 300 less than usual. The year opened with a debit balance of £18 4s. 2d., and closed with a balance in hand of £17 12s. 4d. The council hope, if they receive adequate support from the public, to be able at no distant date to open a small cottage hospital.

---

THE surgeon at Hammersmith who administered a powder to a run-away-ring has found that powder an expensive one; it cost him forty shillings. Run-away-rings are certainly tiresome and irritating to busy men, and to keep the temper with children given to this form of amusement is very difficult. Mr. Alderson took the law into his own hands and administered a medical remedy in the shape of a powder, also saying he had a good mind to draw one of the child's teeth. The story reminds us of the exceedingly practical mother who always gave her children castor-oil when they were naughty, declaring that temper was the outcome of a deranged stomach.

---

THE editor of the *Edinburgh Evening Dispatch* is a great admirer of the dispensaries of that town, and apparently a great foe to missionary enterprise. He says :—"The object of these institutions is a most praiseworthy one, and the benefits which they confer on the sick poor are almost incalculable, somewhere about 40,000 applications for advice or medicine being dealt with in the course of a year, while the total cost amounts to only about one-fifth of what one of our Churches squanders abroad upon Jews who persistently refuse to be converted." Sooner or later missionary charities will have to undergo the scrutiny now given to medical charities; but in the meanwhile surely Edinburgh is rich enough to support both dispensaries and foreign missions.

---

THE unhealthiness of back-to-back houses has been the cause of an inquiry at Liverpool. Dr. Stopford Taylor, the medical officer of health of Liverpool, said a large number of houses in that city had been represented by him under a local act as dangerous to health and unfit for human habitation. This was the first instance in which the owners of property condemned under this act had been able to be represented before the grand jury. The principal objection to these houses was in the fact that they were "back to back," and the argument really turned upon the question whether houses of this character were fit for occupation when erected on a crowded site in a city. The Corporation of Liverpool requested Mr. Shirley Murphy, of London, and Dr. Littlejohn, medical officer of health of Edinburgh, to inspect the houses and state their views to the grand jury. Evidence was given as to death-rates in back-to-back houses and other houses occupied by a similar class of inhabitants, but having through ventilation, with the result that the grand jury determined to recommend that the whole of the houses should be demolished.

THIS is the age of congresses; on every side and in every country we hear of this or that congress, and a large number of them are medical. The late Medical Congress at Melbourne was attended by six or seven hundred doctors; the late Medical Congress in Russia was attended by many men, but also by one hundred and sixty-two medical women. A Surgical Congress will be held at Berlin at Easter; a Congress of Medical Relief will be held at Paris in the end of July; a Congress of Otology will be held at Florence in 1892, under the presidency of Dr. Vittoria Grazzio. Surely in this meeting together of the professors of the healing art we can trace a seed which will help the growth of that vision described by Tennyson in "Locksley Hall":

"Till the war-drum throbb'd no longer, and the battle flags were furl'd,
In the Parliament of man, the Federation of the world."

---

THE fortieth annual meeting of the Royal Portsmouth, Portsea, and Gosport Hospital was held in Portsmouth Guildhall on the 12th ult. Mr. J. Pares, who occupied the chair, said that this had been an eventful year for the hospital. The building had been reconstructed in a satisfactory manner, though the demands on the accommodation are so great that it must soon be further enlarged. A steward has been appointed, and the probationers and outside nurses are doing good work. The in-patients during last year numbered 744, while 6,650 were treated in the out-patient department. There has been a reduction in the average cost per patient, but nevertheless the hospital ends the year with a deficiency of £400, though there have been several handsome donations during the year. Sir William King, the chairman of Committee, mentioned one from an anonymous donor of £1,100. The committee referred in terms of praise to the energy and ability displayed by Miss Tillett, the matron, and alluded to the improvement in the system of nursing which has been effected under her management.

---

AT last we have found the Perfect Report for which we have always longed when wading through the countless different-coloured pamphlets which pile themselves beside the editorial chair! The Halifax Infirmary publishes a report drawn up in such a manner as to furnish all the information that can be looked for, and the accounts of income and expenditure are presented in a perfectly clear and satisfactory manner. The details of expenditure given on pp. 15-17 correspond with the totals in the balance-sheet on p. 12. If similar accounts were furnished in the reports of other hospitals, and especially if particulars similar to those on p. 19 were given by them, there would then be an excellent basis of comparison. Reports should be of value for this purpose, as well as of interest to their own particular supporters. The cost of food and other items of household expenditure show careful administration, especially when taken in conjunction with the fact that the diet throughout the house is an exceedingly liberal one. The greatest difficulties with which the board of management have to contend are the continually overcrowded state of the wards, and also the deficiency of sleeping accommodation for the nurses. These call for an immediate extension of premises, which it is to be hoped the board will see their way to accomplish. One feature in connection with the Halifax Infirmary is deserving of special mention; it is the liberal support given to it by the operatives, amounting last year to £1,782. This is not the result of spasmodic effort; it is the outcome of a very simple but effective organisation, which has been in operation for four years. It is managed entirely by the workmen themselves, who hand the amount collected to the treasurer at the end of the year. There are representative working men of the board of management. Even the lamplighters and scavengers of Halifax periodically make collections amongst themselves for the Infirmary. We advise all hospital secretaries to secure a copy of the Halifax Infirmary Report, and after studying it, to go and do likewise.

